# Optimal Surgical Extent for Memory and Seizure Outcome in Temporal Lobe Epilepsy

**DOI:** 10.1002/ana.26266

**Published:** 2021-11-20

**Authors:** Daichi Sone, Maria Ahmad, Pamela J. Thompson, Sallie Baxendale, Sjoerd B. Vos, Fenglai Xiao, Jane de Tisi, Andrew W. McEvoy, Anna Miserocchi, John S. Duncan, Matthias J. Koepp, Marian Galovic

**Affiliations:** ^1^ Department of Clinical and Experimental Epilepsy University College London Queen Square Institute of Neurology London UK; ^2^ Magnetic Resonance Imaging Unit Epilepsy Society Chalfont St Peter UK; ^3^ Department of Psychiatry The Jikei University School of Medicine Tokyo Japan; ^4^ Centre for Medical Image Computing (CMIC) University College London London UK; ^5^ Neuroradiological Academic Unit, UCL Queen Square Institute of Neurology University College London London UK; ^6^ Department of Neurology, Clinical Neuroscience Center University Hospital Zurich Zurich Switzerland

## Abstract

**Objective:**

Postoperative memory decline is an important consequence of anterior temporal lobe resection (ATLR) for temporal lobe epilepsy (TLE), and the extent of resection may be a modifiable factor. This study aimed to define optimal resection margins for cognitive outcome while maintaining a high rate of postoperative seizure freedom.

**Methods:**

This cohort study evaluated the resection extent on postoperative structural MRI using automated voxel‐based methods and manual measurements in 142 consecutive patients with unilateral drug refractory TLE (74 left, 68 right TLE) who underwent standard ATLR.

**Results:**

Voxel‐wise analyses revealed that postsurgical verbal memory decline correlated with resections of the posterior hippocampus and inferior temporal gyrus, whereas larger resections of the fusiform gyrus were associated with worsening of visual memory in left TLE. Limiting the posterior extent of left hippocampal resection to 55% reduced the odds of significant postoperative verbal memory decline by a factor of 8.1 (95% CI 1.5–44.4, *p* = 0.02). Seizure freedom was not related to posterior resection extent, but to the piriform cortex removal after left ATLR. In right TLE, variability of the posterior extent of resection was not associated with verbal and visual memory decline or seizures after surgery.

**Interpretation:**

The extent of surgical resection is an independent and modifiable risk factor for cognitive decline and seizures after left ATLR. Adapting the posterior extent of left ATLR might optimize postoperative outcome, with reduced risk of memory impairment while maintaining comparable seizure‐freedom rates. The current, more lenient, approach might be appropriate for right ATLR. ANN NEUROL 2022;91:131–144

Anterior temporal lobe resection (ATLR) is the most commonly performed approach for surgical treatment of drug‐refractory temporal lobe epilepsy (TLE), providing a 50–80% chance of seizure freedom.^1^, ^2^ Following ATLR in the language‐dominant, usually left, temporal lobe, there is a risk of verbal memory decline, whereas surgery in the non‐dominant temporal lobe leads less frequently to cognitive problems.^3^, ^4^ Postoperative memory difficulties may impair quality of life^5^, ^6^ and the ability to work or study. Older age at surgery, higher levels of preoperative function, and postoperative seizures are associated with greater risk of impaired cognition after ATLR.^7^, ^8^

The surgical extent and approach vary for ATLR.^9^ It is usually assumed that the extent of surgical resection has opposing effects on cognitive and seizure outcome.^10^, ^11^, ^12^, ^13^ The functional integrity of the posterior hippocampal remnant is thought to support postoperative memory function and so to protect against significant verbal memory decline after language‐dominant ATLR, and visual memory after non‐language‐dominant ATLR.^14^, ^15^, ^16^, ^17^

We analyzed the variability in ATLR extent using automated voxel‐wise analyses and manual numerical measurements to detect areas that are associated with memory decline or unfavorable seizure outcome. From a clinical perspective, we aimed to define resection margins that provide an optimal compromise regarding cognitive outcome while maintaining a high rate of postoperative seizure freedom.

## Methods

### Subjects

From our ongoing single‐center prospective cohort study of long‐term outcome after epilepsy surgery,^2^ we identified consecutive individuals with refractory unilateral TLE, who (1) underwent comprehensive presurgical evaluation followed by standard ATLR, (2) received standardized pre‐ and post‐surgical neuropsychological evaluation, (3) had at least 1 year of postoperative follow‐up, and (4) had a 3D T1‐weighted MRI scan 3–4 months postoperatively between 2004 and 2016. We excluded patients with MRI scans of insufficient quality (ie, patient movement or technical artifacts).

The methods of data extraction and follow‐up were previously described.^2^ In brief, for each individual, diagnosis of TLE was made by a multidisciplinary epilepsy team evaluation involving neurologists, neurophysiologists, neurosurgeons, neuropsychologists, and psychiatrists specializing in epilepsy based on clinical history, neurologic examination results, seizure semiology, long‐term video‐electroencephalography telemetry, MRI, and neuropsychological and psychiatric assessments. Data on language lateralization was available in 91 (64%) of patients who underwent functional MRI (fMRI) test or Wada test. Seizure outcome was assessed annually using a standard surgery outcome classification,^18^ excluding acute seizures within the first postoperative week.

The study was classified by the Institutional Review Board as a service evaluation involving further anonymized analysis of previously acquired data that did not require individual participant consent.

### Standard Neurosurgical Procedure

The standard neurosurgical procedure consisted of identifying the temporal horn entering from the collateral sulcus to minimize damage to the optic radiation and removing the temporal pole en bloc. This was followed by debulking of the amygdala, resection of the piriform cortex and en bloc resection of the hippocampus with a posterior resection margin at the mid‐brainstem level. The resection of the parahippocampal gyrus is also taken to the same level as the hippocampus. All surgeries were conducted by the same neurosurgeon (AWM).

### Neuropsychological Assessment

Patients in our surgical cohort undergo a detailed neuropsychological examination pre‐ and 12‐months post‐operatively. For this study verbal and visual memory was assessed using the List Learning and Design Learning subtests from the BIRT (Brain Injury Rehabilitation Trust) Memory and Information Processing Battery (BMIPB) and its predecessor the AMIPB. These measures are commonly used clinically in the UK and they have been demonstrated to be sensitive to the integrity of temporal structures.^19^

The List Learning Test from the AMIPB/BIMPB battery shares a similar structure to the California Verbal Learning Test. The patient is read aloud a list of 15 words and asked to recall as many words as possible over five trials, providing a maximum score of 75. In the Design Learning Test, the patient is presented with a design comprising of nine connected lines on a 4 × 4 dot matrix for 10 s and the patient is required to reproduce it from memory on a blank grid after each presentation. The total number of correctly placed lines over five trials is recorded, generating a maximum design learning score of 45.

The difference between pre‐ and 1‐year postsurgical learning test scores converted into z‐scores was the indicator of memory change. Significant memory decline was defined as decline on the 80% reliable change index (RCI) as described previously.^7^ The RCI reflects meaningful memory decline adjusted for a test's reliability and practice effects in a test–retest setting.

Additionally, as naming difficulty is a common cognitive dysfunction and can be worsened after temporal lobe surgery,^20^ we also assessed postoperative changes in language function. Patients underwent the Graded Naming Test^21^ pre‐ and 12‐months post‐operatively, and the postoperative score changes were analyzed.

### MRI Acquisition and Processing

Postsurgical MRI scans were performed between January 2004 and August 2013 on a 3T GE Signa HDx Scanner (GE, Milwaukee, WI, USA), at the Epilepsy Society. A coronal T1W 3D fast spoiled gradient echo (FSPGR) with repetition time/echo time/inversion time = 6.6/2.8/450 ms; matrix 256 × 256 × 178; field‐of‐view 24 × 240 × 196 mm; voxel size 0.9375 × 0.9375 × 1.1 mm was acquired in all subjects. Subjects scanned after August 2013 underwent imaging on a 3T GE Discovery MR750. A 3D T1‐weighted inversion‐recovery fast spoiled gradient recalled echo (TE/TR/TI 3.1/7.4/400 ms, field of view (FOV) 224 × 256 × 256 mm, matrix 224 × 256 × 256, parallel imaging acceleration 2) was acquired.

**FIGURE 1:**
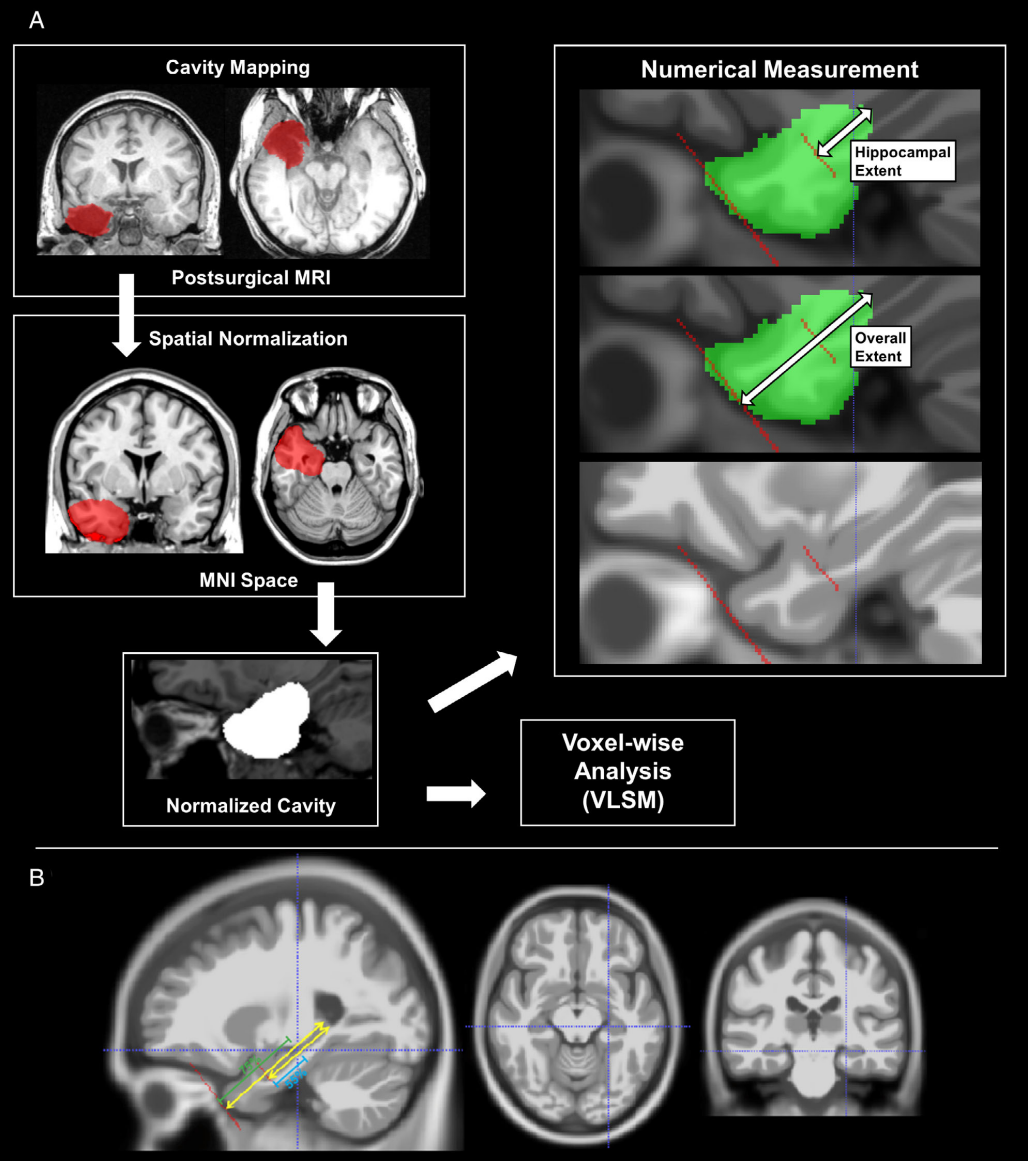
(A) The concept and pipeline of this study. The surgical cavity mask was outlined and spatially normalized. The normalized cavity was investigated by voxel‐wise analysis and numerical measurements. For numerical measurements, we used the tip and tail of the hippocampus, the tip of the temporal lobe and the hippocampal axis as landmarks. A blinded rater measured the length of hippocampal resection in template space as the distance from the most anterior hippocampal voxel to the most posterior resected voxel within the hippocampus. Similarly, we measured the overall temporal lobe resection length as the distance from the most anterior part of the temporal pole to the most posterior resected voxel within the temporal lobe along the hippocampal axis. The relative resection length was calculated as the proportion between the resection length and the overall length of the hippocampus or temporal lobe. (B) Areas around the cut‐off point (55% of hippocampal length and 75% of temporal lobe length) in the numerical analysis. The crosshair denotes the identified cut‐off point for a resection extent with optimal postsurgical outcome.

The surgical cavity was initially automatically segmented using a previously described procedure^22^ with the unaffected contralateral temporal lobe acting as reference. All surgical cavity masks were subsequently checked by an investigator blinded to surgical outcome and, if necessary, manually adjusted (Fig 1, upper left).

We performed spatial normalization of MRI data with Diffeomorphic Anatomical Registration using Exponentiated Lie algebra (DARTEL)^23^ implemented in the Computational Anatomy Toolbox 12 (CAT12, http://www.neuro.uni-jena.de/cat/) running on Statistical Parametric Mapping 12 (SPM12, https://www.fil.ion.ucl.ac.uk/spm/). To reduce the effects of the surgical cavity on spatial normalization we used an enantiomorphic masking approach, that provides accurate spatial transformations for brains with large lesions.^24^

### Voxel‐Based Lesion‐Symptom Mapping (VLSM)

We used non‐parametric mapping (NPM) implemented in MRIcron for voxel‐based lesion‐symptom mapping (VLSM)^25^ of spatially normalized resection cavity masks. VLSM analysis was conducted separately for left and right TLE, along with a separate analysis for both verbal and visual learning changes. The differences of pre‐ vs. postsurgical z‐scores (or score differences for language function) were used as the variables of interest for the VLSM analyses, after correction for age, preoperative memory (or language) function, and seizure outcome. Additionally, the relationship between the resected areas and seizure freedom 1‐year after surgery was analyzed. Clusters with *p* < 0.05 corrected for multiple testing using false discovery rate and a minimum size of 30 contiguous voxels were deemed significant.

### Measurement of Posterior Resection Cavity Extent

We determined the anterior to posterior extent of the resection cavity as a proportion of the length of the hippocampus and the temporal lobe (Fig 1A). Our aim was to analyze the association of the anterior–posterior resection extent with memory outcome and postoperative seizures and to define a maximal resection length that optimizes postsurgical outcome.

Firstly, we defined the tip and tail of the hippocampus, the tip of the temporal lobe and the hippocampal axis as landmarks in template space. We then determined the overall anterior to posterior length of the hippocampus (from the anterior tip to the most posterior tail on the sagittal slices of MNI template) and the temporal lobe (from the tip of the temporal pole to the tail of the hippocampus). Next, we measured the length of hippocampal resection in template space as the distance from the most anterior hippocampal voxel to the most posterior resected voxel within the hippocampus, as outlined in Fig 1. We repeated the same procedure for the overall temporal lobe, determining the distance from the most anterior part of the temporal pole to the most posterior resected voxel within the temporal lobe, measuring along the hippocampal axis. Lastly, we calculated the relative resection length as the proportion between the resection length and the length of the hippocampus or temporal lobe. All measurements were manually performed by a rater blinded to postoperative outcome.

### Numerical Analysis for Optimal Resection Extent

We used the area under the receiver operating characteristic curve (AUC) to measure the association between resection length with postoperative verbal or visual memory decline defined by RCIs and with postoperative seizure outcome.

To inform the optimal extent of the posterior surgical resection boundary, we determined cut‐offs by examining AUCs at different hippocampal and temporal lobe resection lengths, supported by a visual analysis of scatter plots of resection extent and postoperative outcome. We examined odds ratios (OR) for cognitive decline or postoperative seizures at the selected cut‐offs using logistic regression and adjusted the results for age, presurgical memory performance, and postsurgical seizures. Statistical analyses were done in SPSS (version 26, IBM Corp.).

### Preoperative Hippocampal Atrophy and Resection Extent

To elucidate a potential bias for larger resection extent due to preoperative hippocampal tail atrophy, we analyzed preoperative 3D‐T1W FSPGR images using a hippocampal profiling tool.^26^ Hippocampal profiling is designed to segment and analyze the hippocampus in people with epilepsy. It calculates the hippocampal volume corrected for total intracranial volume as well as the hippocampal cross‐sectional area (CSA) along the hippocampal axis.^26^ First, we performed correlation analysis between the calculated ipsilateral hippocampal volumes and the numerical resection extent. Additionally, we divided the patients into groups with or without hippocampal tail atrophy. We defined hippocampal tail atrophy as a significantly reduced CSA at a ≥ 35 mm distance from the anterior tip of hippocampal head. The differences in numerical hippocampal resection extent between groups with (+) vs. without (−) hippocampal tail atrophy were also analyzed.

## Results

We included 142 eligible patients with TLE (74 left) in the final analysis. The demographic and clinical features of the patients are presented in Table 1. There was missing data on visual learning in three patients and, thus, visual learning was analyzed in the available data of 139 patients.

**TABLE 1:** Demographics of Patients with Left and Right TLE

	Left TLE(n = 74)	Right TLE(n = 68)
Demographics		
Age at surgery (yr)	36.7 ± 11.8	37.4 ± 11.2
Gender (M:F)	31:43	34:34
Onset age (yr)	12.2 ± 9.6	14.1 ± 10.5
Right‐handed ^†1^	n = 61	n = 56
Dominant hemisphere (N)	L: 49, R: 2, Bil: 6, N/A: 17	L: 42, R: 3, Bil: 7, N/A: 16
Seizure and drugs before surgery		
Number of AEDs	2.59 ± 1.05	2.62 ± 0.88
Patients with FAS	n = 45	n = 35
Patients with FBTCS	n = 57	n = 50
Neuropsychology		
Verbal IQ ^†2^	90.8 ± 12.2	92.8 ± 14.2
Performance IQ ^†3^	100.1 ± 13.8	94.9 ± 14.9
Verbal learning (Pre‐OP)	−1.04 ± 1.13	−0.65 ± 1.05
Verbal learning (Post‐OP)	−1.46 ± 1.20	−0.49 ± 1.13
Visual learning (Pre‐OP) ^†4^	0.04 ± 1.18	−0.11 ± 0.88
Visual learning (Post‐OP) ^†4^	−0.08 ± 0.83	−0.22 ± 0.86
Language function (Pre‐OP) ^†5^	13.7 ± 4.4	17.1 ± 5.0
Language function (Post‐OP) ^†5^	12.4 ± 4.9	18.3 ± 4.9
Surgery		
Seizure free (Engel I)	N = 40 (54%)	N = 37 (54%)
Preoperative MRI	HS: 56, Normal: 7, Others: 11	HS: 42, Normal: 6, Others: 20
Pathology	HS: 57, DNT:4, DUAL: 4, Others: 9	HS: 45, DNT: 8, DUAL: 4, Others: 11

Data given as n (%) or mean ± standard deviation.

*Dominant hemisphere was determined by functional MRI or Wada test.

*Seizure and cognitive outcomes were estimated at 1‐year after ATL.

*Values of learning ability are shown in Z‐scores.

*Language functions are shown in scores of the Graded Naming Test.

†1 Unknown in five.

†2 Unknown in six.

†3 Unknown in 20.

†4 Unknown in three.

†5 Unknown in nine.

AEDs = anti‐epileptic drugs; Bil = bilateral = N/A = not available; DNT = dysembryoblastic neuroepithelial tumor; DUAL = dual pathology; F = female; FAS = focal aware seizures; FBTCS = focal‐to‐bilateral tonic–clonic seizures; FIAS = focal impaired‐awareness seizures; HS = hippocampal sclerosis; IQ = intelligence quotient; L = left; M = male; Post‐OP = post‐operative; Pre‐OP = pre‐operative; R = right.

Based on RCIs, in left TLE, 24 patients (32%) showed significant verbal memory decline, while visual memory declined significantly in 16 patients (22%). In right TLE, we found 12 patients (18%) with significant verbal memory decline, and seven patients (11%) with significant visual memory decline. There was a nonsignificant trend for worse postoperative verbal memory outcome in people with left‐dominant language lateralization after left ATLR and with atypical language lateralization after right ATLR (Table 2). Additionally, postoperative memory outcome did not significantly differ in association with the existence of hippocampal sclerosis (Table 2). All patients took the same antiseizure drugs at 1‐year after surgery as at surgery.

**TABLE 2:** Postsurgical Memory Changes between Patients with Left and Atypical Language Dominance

	Change in verbal learning		
	Left dominance	Atypical pattern	*p*‐value
Left TLE	−0.61 ± 1.00	0.18 ± 1.41	0.055
Right TLE	0.24 ± 0.91	−0.32 ± 0.96	0.088
	HS + DUAL	Non‐HS	
Left TLE	−0.35 ± 1.04	−0.69 ± 0.89	0.323
Right TLE	0.12 ± 1.02	0.26 ± 0.92	0.594
	**Change in visual learning**		
	Left dominance	Atypical pattern	*p* Value
Left TLE	−0.26 ± 1.27	−0.38 ± 0.82	0.786
Right TLE	−0.02 ± 0.69	−0.05 ± 0.78	0.920
	HS + DUAL	Non‐HS	
Left TLE	−0.05 ± 1.13	−0.44 ± 1.34	0.280
Right TLE	−0.07 ± 0.71	−0.09 ± 0.73	0.910

*Values of learning ability are shown in Z‐scores, and higher Z‐scores denote better postsurgical outcome.

*Atypical pattern includes patients with right or bilateral language dominance.

*p‐values were calculated by unpaired *t*‐test.

### Extent of Neurosurgical Resection

The mean anterior–posterior extent of the resection was 79% ± 8% and 81% ± 11% of the distance from the temporal pole to the posterior tip of the hippocampal tail after left and right anterior temporal lobe resection, respectively. Within the hippocampus, the mean resection extent affected 61% ± 14% of the anterior–posterior length of the hippocampus after left and 67% ± 19% after right anterior temporal lobe resection.

### Voxel‐wise Analysis

**FIGURE 2:**
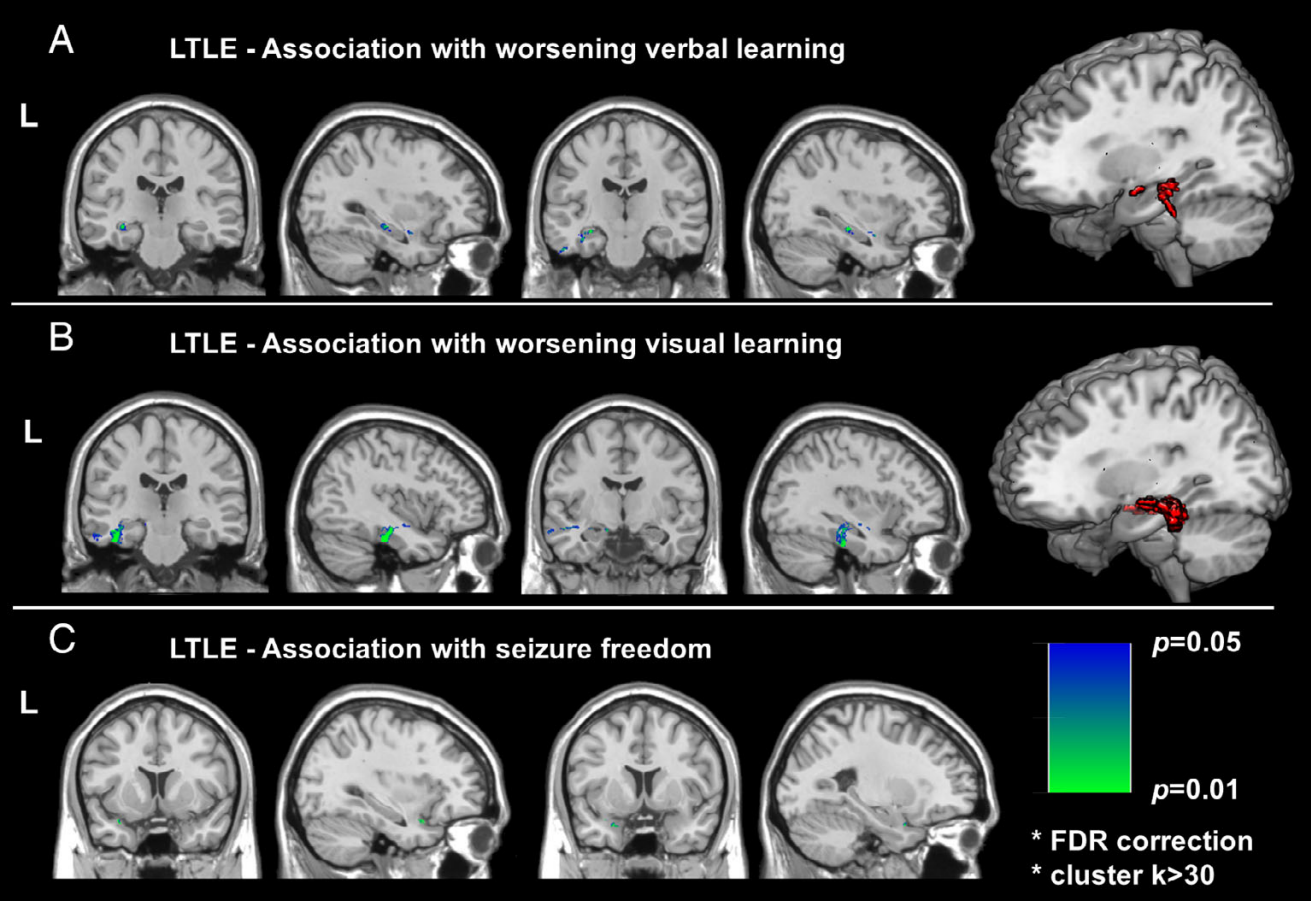
Voxel‐wise association with postoperative memory and seizure freedom in left TLE. Significantly correlated areas with (A) verbal memory (B) visual memory, and (C) seizure freedom. Red voxels in the right denote significant clusters at FDR *p* < 0.05 and cluster k > 30 by VLSM statistics.

In left TLE, we detected an association of posterior hippocampal resection with postsurgical verbal memory decline (Fig 2A). Resection of the inferior temporal gyrus was also associated with verbal memory decline (Fig 2A). Resection of the fusiform gyrus was associated with visual learning decline, in addition to the upper temporal white matter and hippocampus (Fig 2B). In terms of seizure outcome, a greater extent of piriform gyrus resection was significantly associated with postoperative seizure freedom (Fig 2C).

In right TLE, resection variability was not associated with postoperative verbal and visual memory decline or seizure freedom.

### Numerical Analysis

**FIGURE 3:**
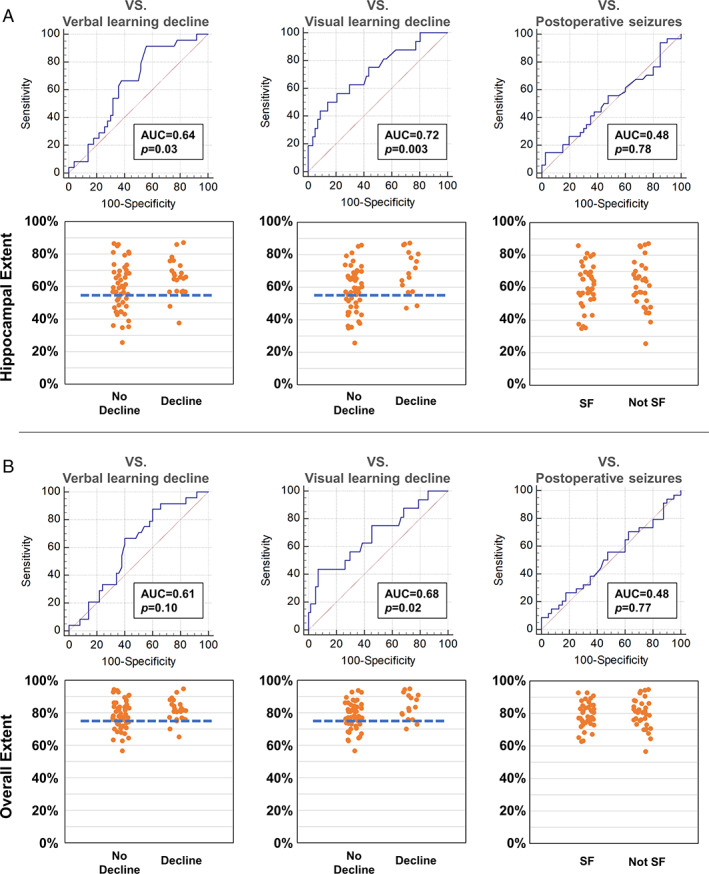
Posterior extent of resection and memory and seizure outcomes after left ATLR. Areas under the receiver operating characteristic curve and scatter plot for hippocampal extent (A) and overall temporal lobe extent (B).

The results of numerical indicators of resection extent in left TLE are shown in Figure 3. Hippocampal resection extent variability was associated with both verbal (AUC 0.64, *p* = 0.03) and visual (AUC 0.72, *p* = 0.003) memory decline in left TLE. The overall extent of temporal lobe resection was associated with visual (AUC 0.68, *p* = 0.02) but not verbal (AUC 0.61, *p* = 0.10) memory decline. Postsurgical seizure outcome was not associated with hippocampal (AUC 0.48, *p* = 0.78) nor overall (AUC 0.48, *p* = 0.77) resection extent variability.

**FIGURE 4:**
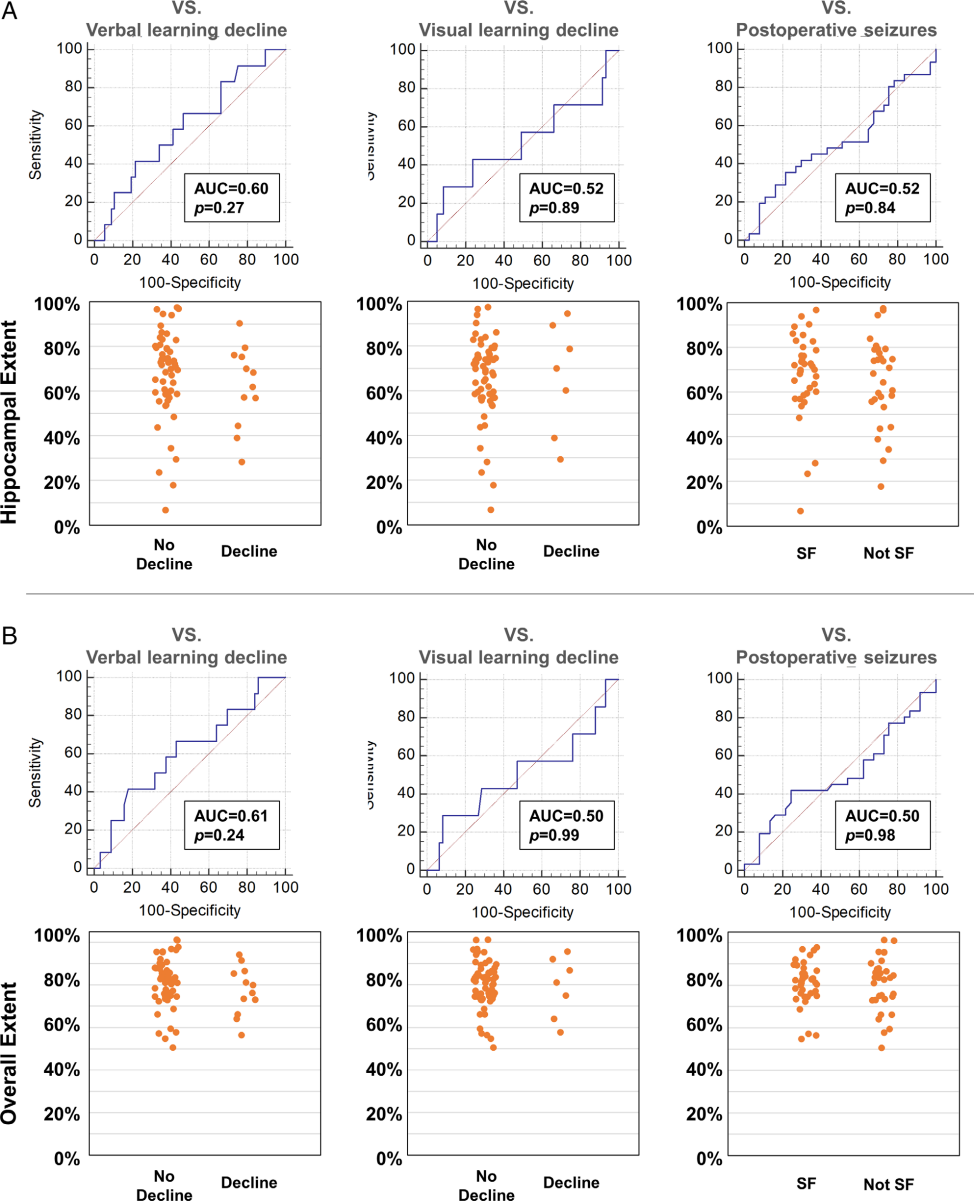
Posterior extent of resection and memory and seizure outcomes after right ATLR. Areas under the receiver operating characteristic curve and scatter plot for hippocampal extent (A) and overall temporal lobe extent (B).

In right TLE, the posterior extent of hippocampal and overall resection was not associated with memory decline or postoperative seizures (Fig 4).

### Optimization of Posterior Resection Extent

To define cut‐offs that optimize memory outcome while maintaining a high rate of seizure‐freedom after left ATLR we determined AUCs for different hippocampal and temporal lobe resection lengths and visually assessed the scatter plots of hippocampal and overall temporal lobe resection extent (Fig 3). We chose a 55% posterior resection extent of the anterior to posterior hippocampal length as an optimal maximal cut‐off within the hippocampus. For the overall resection extent, we defined 75% of the distance from the tip of the temporal pole to the tip of the hippocampal tail as an optimal maximal cut‐off. The area around this cut‐off point is also presented in Figure 1B. The identified optimal resection extent extends to around the midline of the brainstem in template space.

Limiting the posterior extent of left hippocampal resection to a maximum 55% of the anterior–posterior hippocampal length (Fig 1B) reduced the odds of significant postoperative verbal memory decline from 41% to 10% by a factor of 8.1 (95% CI 1.5–44.4, *p* = 0.02, Table 3A). Resecting ≤55% of the hippocampal length was associated with a nonsignificant reduction of postsurgical significant visual memory decline from 26% to 11% (OR 4.3, 95% CI 0.7–25.6, *p* = 0.11) and only minimal differences in postoperative seizure outcome (OR 0.8, 95% CI 0.3–2.2, *p* = 0.79, Table 3A).

**TABLE 3:** Posterior Resection Extent and Postsurgical Memory and Seizures in Left TLE

	Smaller resection	Larger resection	OR (95% CI)	*p*‐value
A. Hippocampal resection extent smaller or bigger than 55%				
Verbal memory decline	2 (10%)	22 (41%)	8.1 (1.5–44.4)	0.02
Visual memory decline	2 (11%)	14 (26%)	4.3 (0.7–25.6)	0.11
Postoperative seizures	10 (50%)	24 (44%)	0.8 (0.3–2.2)	0.79
B. Temporal lobe resection extent smaller or bigger than 75%				
Verbal memory decline	2 (11%)	22 (40%)	6.1 (1.2–31.6)	0.03
Visual memory decline	2 (11%)	14 (26%)	4.1 (0.7–25.1)	0.12
Postoperative seizures	9 (47%)	25 (46%)	0.9 (0.3–2.6)	1.00

Limiting the posterior extent of left temporal lobe resection to a maximum 75% of the distance from the tip of the temporal pole to the tip of the hippocampal tail (Fig 1B) reduced the odds of significant postoperative verbal memory decline from 40% to 11% (OR 6.1, 95% CI 1.2–31.6, *p* = 0.03, Table 3B). Resecting ≤75% of the temporal lobe was associated with a nonsignificant reduction of postsurgical visual memory decline from 26% to 11% (OR 4.1, 95% CI 0.7–25.1, *p* = 0.12) and no differences in postoperative seizure outcome (OR 0.9, 95% CI 0.3–2.6, *p* = 1.00, Table 3B).

Visual memory decline was associated with large resections. If >75% of left hippocampus was resected, the odds of visual memory decline were increased by a factor of 11.8 (95% CI 2.2–63.3, *p* = 0.004). Similarly, the odds of visual memory decline if >90% temporal lobe was resected were increased by a factor of 8.6 (95% CI 1.3–55.1, *p* = 0.02).

In RTLE, the variability of the extent of hippocampal or temporal lobe resections were not associated with verbal and visual memory decline or seizures after surgery and optimal cut‐offs could not be defined (Fig 4).

### Sensitivity Analysis

**FIGURE 5:**
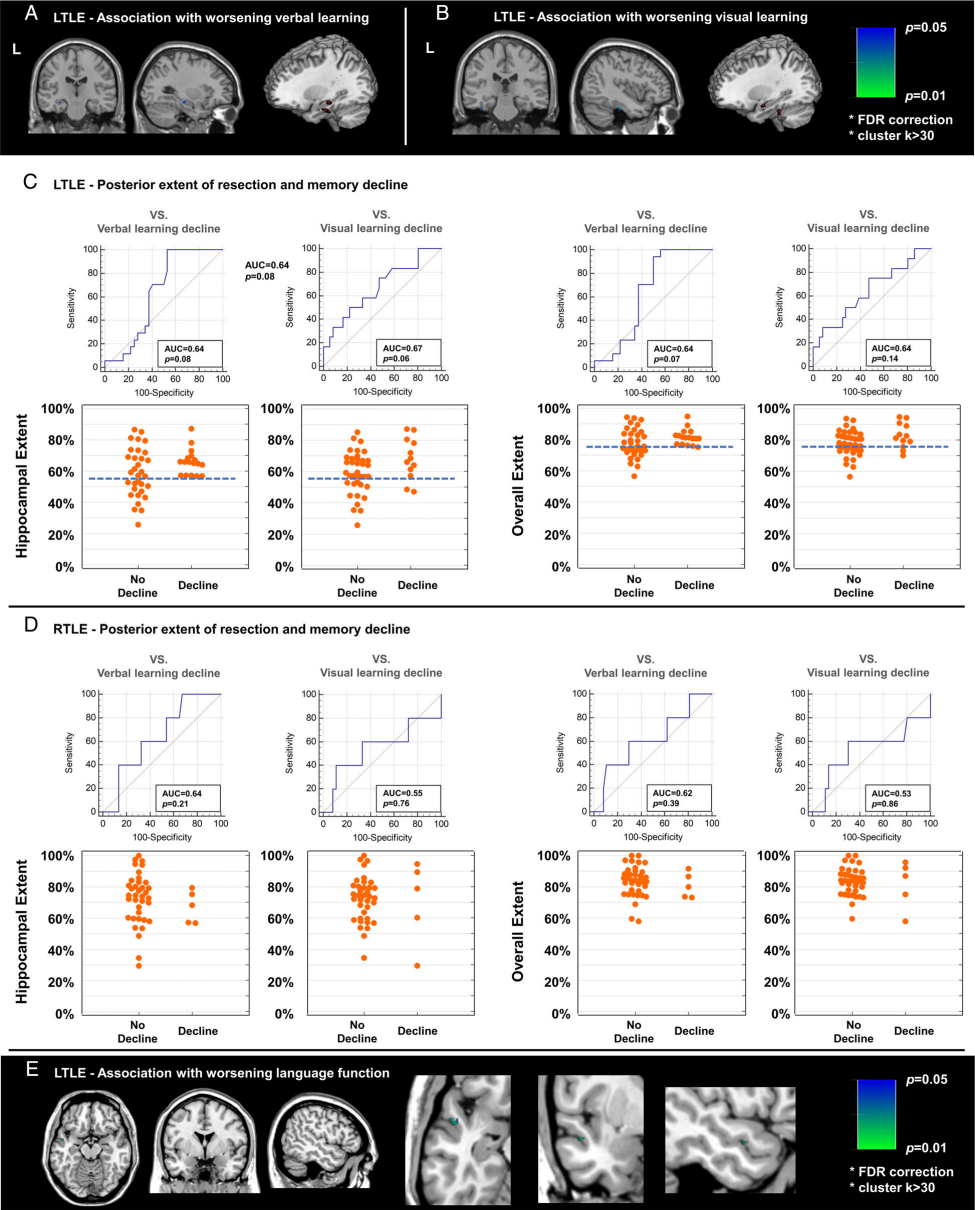
Results of subanalysis using only left language‐dominant patients. (A–B) Voxel‐wise association with postoperative verbal (A) and visual (B) memory in left TLE. (C‐D) Posterior extent of resection and memory and seizure outcomes after left (C) and right (D) ATLR. Voxel‐wise association with postoperative language function in left TLE was also found (E).

To account for language dominance, we performed the same analysis on a smaller sample of patients (49 left and 42 right TLE) with left language dominance confirmed by fMRI or Wada test. Similar results were obtained in relation to posterior extent for hippocampal and overall resection, as well as voxel‐wise analysis, albeit at a lower level of significance (Fig 5).

### Preoperative Hippocampal Atrophy and Resection Extent

Preoperative 3D‐T1W images were available in 82 patients (40 LTLE and 42 RTLE). Pearson's correlation analysis revealed no significant association between preoperative hippocampal volumes and hippocampal surgical extent in LTLE (r = −0.013, *p* = 0.937) and RTLE (r = −0.077, *p* = 0.628). Based on hippocampal profiling along the anterior–posterior axis, there were 41 patients with (28 LTLE, 13 RTLE) and 41 without (12 LTLE, 29 RTLE) hippocampal tail atrophy. There was no significant difference in the hippocampal resection extent between these two groups (*p* = 0.156 in LTLE, *p* = 0.755 in RTLE).

There was no association between preoperative hippocampal tail atrophy and seizure outcome for the whole cohort (*p* = 0.817, χ^2^ test). However, when analyzing the subgroup of LTLE with preoperative hippocampal tail atrophy, patients with seizure freedom had a larger hippocampal resection extent than those with ongoing seizures (62.7 ± 10.5% vs 49.1 ± 13.2% resection, *p* = 0.007, unpaired t‐test). This suggests that larger resections of the left hippocampus may be needed to achieve seizure freedom in cases with hippocampal tail atrophy. No such tendency was found in LTLE without hippocampal tail atrophy nor RTLE.

### Language Function and Surgical Extent

We also analyzed postoperative changes in language function and the associated resection areas. As shown in Table 1, patients with LTLE experienced a decline of language function (*p* = 0.002, paired t‐test), while RTLE showed improved language function (*p* < 0.001, paired t‐test) on average. On VLSM analysis, resections involving a small area in the left superior temporal gyrus were significantly associated with worsening of postoperative language function in the left language‐dominant patients with LTLE (Fig 5E). There were no significant clusters when analyzing the combined group of LTLE and RTLE patients.

## Discussion

We investigated the association of the extent of ATLR with memory and seizure outcome in TLE. In left TLE, postsurgical verbal memory decline was correlated with resection of the posterior hippocampus and inferior temporal gyrus, whereas resection of the fusiform gyrus was associated with worsening of visual learning. A reduced risk of postoperative verbal and, to a lesser extent, visual memory decline with comparable seizure freedom rates would be achieved by restricting resection extent in left TLE to a maximum 55% of the hippocampal length and 75% of temporal lobe length. The risk of memory difficulties was lower after right compared to left‐sided resections (verbal memory decline LTLE 32% vs. RTLE 18%; visual memory decline LTLE 22% vs. RTLE 11%). These observations may be important to optimize the length of left ATLR to achieve favorable memory and seizure outcome. The variability of the resection extent in right TLE did not correlate with memory decline or postoperative seizure outcome, thus supporting the current more lenient surgical approach in the right hemisphere.

Our results are in line with suggestions that the integrity and amount of temporal lobe tissue removed during surgery contributes to postoperative memory deficits. A larger hippocampal remnant after surgery in left TLE was associated with better verbal memory in previous studies.^27^, ^28^, ^29^ Another study suggested the hippocampal integrity as a relevant factor for postoperative verbal memory in left TLE.^30^ Verbal memory encoding fMRI studies indicated that activation in the ipsilateral posterior hippocampus pre‐operatively supports verbal memory following left hippocampal resection, and activation of the posterior right hippocampus protects visual memory on a visual memory fMRI task.^14^, ^15^ Regarding the physiological memory system, the anterior and posterior parts of hippocampus along the long axis have both shared and differing connectivity with other brain regions.^31^, ^32^ Particularly, considering the connection between the posterior hippocampus and parieto‐occipital cerebral regions,^32^ preserving the posterior hippocampus appears to be important to mitigate memory decline after ATLR.

In contrast, seizure freedom rates did not correlate with the variability of posterior ATLR extent on voxel‐wise and numerical analyses. These observations have important practical implications. Neurosurgeons may consider restricting the posterior extent of hippocampal and temporal neocortical resection to preserve brain tissue that is critical for postoperative cognitive function. We demonstrated that limiting the posterior extent of left‐sided resections to below 55% of the hippocampal length and below 75% of the temporal lobe length may lead to comparable seizure‐freedom rates while minimizing verbal and visual memory decline. Such an imaging‐guided approach may help improve postoperative outcome. However, cases with preoperative left hippocampal tail atrophy might need larger resections to achieve postoperative seizure freedom. Currently, a detailed case by case analysis of the extent of hippocampal atrophy, the size of hippocampal resection and their impact on seizure outcome is underway.

Resection of areas in the temporal neocortex may also influence memory outcome. Our voxel‐wise analysis showed that verbal memory decline after left ATLR was associated with resection of areas in the lateral temporal lobe, particularly the inferior temporal gyrus (Fig 2A). Accordingly, previous studies found that patients with lateral temporal lesions sparing the hippocampus had deficits in immediate and delayed verbal recall.^33^, ^34^ Neurons in the lateral temporal lobe are activated during memory encoding for a wide array of stimuli^35^ and during word encoding tasks in left TLE.^16^ Taken together, the lateral temporal lobe plays a role in a widespread network underlying memory encoding and its resection or disconnection may have adverse effects on this function.

Visual memory decline after left ATLR was associated with larger resections involving the fusiform gyrus (Fig 2B). The inferior temporal cortex plays a central role in the ventral visual pathway implicated in object, face and scene perception.^36^ The fusiform gyrus in particular is involved in higher processing of visual information and object recognition,^37^ and in picture naming.^38^ The left fusiform gyrus is part of the network activated during visual encoding of faces in left TLE.^16^ A smaller resection extent and preservation of these areas might reduce the risk of postsurgical visual memory decline. These findings also underline the observation that visual memory is not strictly lateralized and deficits in visual memory can arise after both left and right temporal lobe removal.^39^

Corroborating previous findings,^22^ postoperative seizure‐freedom in left TLE was associated with resection of the piriform cortex (Fig 3). The piriform cortex is an area highly susceptible to epileptogenic stimulation.^40^ Resection of the piriform cortex may, thus, effectively disrupt the epileptic network in the temporal lobe and interrupt connections between the temporal and frontal cortex, preventing spread of epileptic activity and so leading to higher seizure‐freedom rates.^22^

We did not find an association of resection variability with neither memory nor seizure outcome after right‐sided surgery. Together with the association between visual decline and extent of left‐sided resections, our findings are in keeping with several recent reports that the dogma of material‐specific lateralization does not hold true for many cases.^39^, ^41^ Neocortical resections in ATLR are generally larger in the non‐speech‐dominant hemisphere as there is less concern of damaging language networks.

Naming difficulty is another common dysfunction in TLE,^20^ and most of the relevant brain regions for naming are located in anterior temporal cortex or occipital‐temporal junction.^42^ We found that resections involving the anterior part of the left superior temporal gyrus were associated with postsurgical worsening of naming function in left language‐dominant patients with LTLE. This confirms the relevance of this area for language function and highlights the potential importance of preserving lateral temporal areas to maintain language abilities following surgery. Notably, the basal temporal language area was not correlated with postsurgical naming worsening. According to the literature,^43^ electrical stimulation on the fusiform gyrus 3–7 cm from temporal pole induced language interference, but resection of this area did not cause persistent language deficit.

Our study has limitations. First, our results apply to ATLR only and cannot be directly translated to other techniques, such as selective amygdalohippocampectomy^44^ or laser interstitial thermal therapy.^45^ Our findings provide a proof of concept for a methodology to optimize the planning and extent of selective surgical techniques, that warrant further study.

Second, the predictive value of posterior resection extent for postoperative memory decline is moderate (Fig 3). Other factors play a role, including the strength of anterior hippocampal activation on verbal memory encoding fMRI, age at surgery, the level of preoperative cognitive function, and postoperative seizures.^7^, ^8^, ^14^ Since other contributing factors cannot be controlled, the present findings are meaningful in terms of providing a means to improve postsurgical outcome at an individual level. Additionally, our voxel‐wise analysis included age, preoperative memory function and seizure outcome as covariates, and thus the results could be generalizable to all subjects receiving ATLR.

Third, the data were collected in a single center. Although we used a standard surgical approach and standardized neuropsychological test, external validation is necessary to confirm the generalizability of the findings.

Fourth, we combined different etiologies of TLE in our cohorts. Although this might have increased data variability, it makes our findings applicable to all types of TLE undergoing ATLR.

Fifth, we combined patients with different language‐dominance patterns. However, a sub‐analysis using only left‐dominant patients (49 left TLE, 42 right TLE) found compatible results (Fig 5). Although we also included patients with various etiologies, including hippocampal sclerosis, the memory outcome did not significantly differ between those with and without hippocampal sclerosis (Table 2). However, since the number of patients who pathologically showed no hippocampal sclerosis were relatively small in left TLE (n = 13), caution should be made for our statistical insignificance and cases without hippocampal sclerosis, given the evidence for higher risk of memory decline in MRI‐negative TLE.^46^

Sixth, we could not fully confirm that the variability of surgical resections observed in this study was caused only by chance. The operations were performed by a single surgeon using a standardized landmark‐based approach. The overall variability of resections observed in our study was small and may have been imperceptible by the surgeon, thus allowing random assortment on a range. On the other hand, there may have been specific factors, conscious or unconscious, that may not have been identified and may have led to a variation of the standard ATLR procedure. With this in mind, we excluded an effect of preoperative hippocampal volume or hippocampal tail atrophy on the extent of resection in a subset of our cohort. In other words, the surgeon was not more likely to resect more posterior areas of the temporal lobe and hippocampus if there was preoperative atrophy of the hippocampal tail. We also corrected our findings for age, seizure outcome, and preoperative function to reduce confounding.

Seventh our analyses were restricted only to areas that showed sufficient variability in resection extent, i.e. the most posterior and superior margins of the resection. Thus, we could not determine whether areas without sufficient variability, i.e. those structures that are always resected during an ATLR, could be spared without increasing the risk of postoperative seizures.

In conclusion, we describe the extent of surgical resection as an independent and modifiable risk factor for cognitive decline and seizures after left ATLR. A more posterior extent of resection leads to higher risk of memory difficulties but does not confer better seizure outcome after surgery. Higher seizure‐freedom rates were associated with resection of the piriform cortex. Based on these findings, left ATLR could be refined to resect the piriform cortex while preserving the posterior hippocampus and temporal neocortex. Posterior extent of right ATLR did not relate to postsurgical neuropsychological outcome, warranting a more lenient surgical approach in the non‐speech‐dominant hemisphere.

## Author Contributions

M.G., M.J.K. and J.S.D. contributed to the conception and design of the study; M.G., D.S., M.A., J.D.T., F.X., S.B.V., A.W.M., A.M., P.J.T. and S.B. contributed to the acquisition and analysis of data; M.G., D.S., M.J.K. and J.S.D. contributed to drafting the text and preparing the figures.

## Potential Conflicts of Interest

Nothing to report.
